# Evaluation of respiratory complications in a cohort of preterm infants who did not receive palivizumab monoclonal antibodies

**DOI:** 10.1590/0034-7167-2021-0362

**Published:** 2022-10-03

**Authors:** Poliana Castro de Resende Bonati, Maria Cândida de Carvalho Furtado, Débora Falleiros de Mello, Paula Carolina Bejo Wolkers, Gabriel de Oliveira Faria, Daniela Marques de Lima Mota Ferreira

**Affiliations:** I Universidade de São Paulo. Ribeirão Preto, São Paulo, Brazil; II Universidade Federal de Uberlândia. Uberlândia, Minas Gerais, Brazil

**Keywords:** Infant, Premature, Respiratory Syncytial Viruses, Palivizumab, Respiratory Tract Diseases, Neonatal Nursing., Recién Nacido Prematuro, Virus Sincitiales Respiratorios, Palivizumab, Enfermedades Respiratorias, Enfermería Neonatal., Recém-Nascido Prematuro, Vírus Sinciciais Respiratórios, Palivizumabe, Doenças Respiratórias, Enfermagem Neonatal.

## Abstract

**Objectives:**

to analyze the occurrence of respiratory complications over the first year of life in preterm infants who did not receive palivizumab monoclonal antibodies.

**Methods:**

analytical retrospective cohort study with preterm infants born between 2012 and 2016 in Uberlândia, state of Minas Gerais, Brazil. Data collection occurred from January to November 2018, by consulting hospital and primary healthcare medical records. Data were processed with the Poisson regression model, with p<0.05.

**Results:**

of a total of 5,213 preterm births, 504 (9.7%) met the inclusion criteria. The preterm infants in this subset were assisted 2,899 times in primary care, which resulted in 1,098 (37.5%) medical diagnoses, of which 803 (78.5%) involved the respiratory tract. Preterm babies fed on formula milk at hospital discharge had more diagnoses of respiratory diseases. Maternal age (p=0.039), respiratory diagnosis at hospital discharge (p=0.028), and number of sporadic appointments (p<0.001) showed a significant association with bronchiolitis; number of sporadic appointments showed a significant association with occurrence of respiratory diseases; and breastfeeding had a protective effect against the development of bronchiolitis.

**Conclusions:**

preterm infants who did not receive palivizumab showed a high percentage of respiratory diseases, and breastfeeding helped protect them against bronchiolitis. It is recommended that these preterm babies be monitored in primary health care.

## RESUMO

**Objetivos:**

investigar intercorrências respiratórias no primeiro ano de vida de prematuros não contemplados com anticorpo monoclonal palivizumabe.

**Métodos:**

estudo de coorte retrospectivo, analítico, com prematuros nascidos entre 2012 e 2016, em Uberlândia, MG. Coleta realizada de janeiro a novembro de 2018, em prontuários hospitalar e da atenção básica. Utilizou-se modelo de regressão de Poisson; considerou-se p<0.05.

**Resultados:**

de 5.213 nascimentos prematuros, 504 (9,7%) atenderam os critérios; esses prematuros tiveram 2.899 atendimentos na atenção básica; 1.098 (37,5%) com diagnóstico médico, 803 (78,5%) do trato respiratório. Prematuros com fórmula láctea na alta hospitalar tiveram mais diagnósticos de doenças respiratórias. Idade materna (p=0,039), diagnóstico respiratório na alta (p=0,028), número de consultas eventuais (p<0,001) apresentaram associação significativa com bronquiolite. Consultas eventuais foram significantes para doenças respiratórias; o aleitamento materno mostrou-se protetor para bronquiolite.

**Conclusões:**

prematuros sem palivizumabe apresentaram elevado percentual de doenças respiratórias; observou-se efeito protetor do aleitamento materno. Recomenda-se vigilância desses prematuros na atenção primária.

## RESUMEN

**Objetivos:**

investigar complicaciones respiratorias en el primer año de vida de prematuros no contemplados con anticuerpo monoclonal palivizumab.

**Métodos:**

estudio de cohorte retrospectivo, analítico, con prematuros nacidos entre 2012 y 2016 en Uberlândia, MG. Datos recolectados de enero a noviembre de 2018 en historias clínicas hospitalarias y de atención básica. Se utilizó modelo de regresión de Poisson, considerándose p<0,05.

**Resultados:**

de 5.513 nacimientos prematuros, 504 (9,7%) atendieron criterios; estos prematuros recibieron 2.899 consultas en atención básica; 1.098 (37,5%) con diagnóstico médico; 803 (78,5%) del tracto respiratorio. Prematuros con fórmula láctea al alta hospitalaria recibieron más diagnósticos de enfermedades respiratorias. Edad materna (p=0,039), diagnóstico respiratorio al alta (p=0,028), cantidad de consultas eventuales (p<0,001) mostraron asociación significativa con bronquiolitis.

**Conclusiones:**

prematuros sin palivizumab mostraron elevado porcentaje de enfermedades respiratorias; se observó efecto protector de la lactancia materna. Se recomienda el seguimiento de dichos prematuros en atención primaria.

## INTRODUCTION

Acute respiratory infections (ARIs) remain the most common cause of morbimortality worldwide, especially in children under five years old^(1-2)^. One of the viruses that causes ARIs is the respiratory syncytial virus (RSV), which is most prevalent in children. Most of the affected children are infected with it up to 12 months of age and nearly all of them up to two years old^(3-5)^. The respiratory syncytial virus is the main cause of bronchiolitis and pneumonia and is related to disease outbreaks and simultaneous infections involving respiratory conditions around the world^(6)^. Studies have indicated its relationship with asthma aggravation, wheezing, and invasive pneumococcal disease^(5,7)^. Additionally, it is the leading cause of child mortality^(5)^.

Preterm infants are even more vulnerable to severe infections caused by RSV, since this population has an immature immune system and a low level of maternal antibodies. Therefore, they need to be admitted to intensive care units and receive invasive ventilatory support more frequently^(4,6-7)^. Bronchiolitis resulting from infection with RSV is an important cause of morbimortality associated with ARIs in preterm infants and the most common cause of hospital admission among newborns worldwide^(4,8-10)^.

Given that a vaccine against RSV has not yet been developed for humans, palivizumab monoclonal antibody (PVZ) is recommended for preventing the severe form of the disease in high-risk children, such as preterm infants, especially those born before 35 weeks of gestational age (GA)^(11)^. Because it is an expensive product, in Brazil, the Ministry of Health has made prophylaxis with PVZ available for children born before 28 weeks of GA since 2013^(11-12)^. Preterm infants with GA over 28 weeks are not covered by the PVZ preventive administration protocol, despite also being susceptible to the severe infections caused by RSV. However, it has been shown that breastfeeding has a protective effect on preterm infants against the development of these infections^(13-14)^.

The evidence above, together with the fact that newborns with moderate prematurity do not receive PVZ^(11)^, motivated the present study. The authors advocate monitoring this population throughout their first year of life, since these babies have higher risks of developing diseases in the respiratory system^(8-9)^, are more susceptible to lower respiratory tract infections, and can undergo more episodes of hospitalization^(6-13)^ and death^(5)^. Therefore, the present study is innovative in bringing together aspects that other studies have considered individually: analysis of preterm infants from birth up to one year; their vulnerability to respiratory complications over this period; and the fact of being born with a GA that is not considered eligible for PVZ administration. The results are expected to be useful for informing nurses so they can provide qualified care oriented toward reducing the incidence of respiratory problems, worsening of these diseases, and hospitalization and death rates in the affected children.

## OBJECTIVES

To analyze the occurrence of respiratory complications over the first year of life in preterm infants who did not receive PVZ monoclonal antibodies.

## METHODS

### Ethical aspects

The proposal was approved by the Research Ethics Committee of the Ribeirão Preto College of Nursing at University of São Paulo as per Certificate of Presentation of Ethical Evaluation and met the guidelines established in National Health Council Resolution no. 466, of December 12, 2012. As the study involved collection of secondary data only, it was exempted from application of free and informed consent forms.

### Study design, location, and period

This was an observational analytical retrospective cohort study carried out in accordance with the Strengthening the Reporting of Observational Studies in Epidemiology checklist. Its setting was a public hospital that is considered one of the most important institutions regarding high-complexity care in the Triângulo do Norte macro region, in the state of Minas Gerais, and primary healthcare services in Uberlândia, in the same state. Births of preterm infants between 2012 and 2016 at this hospital and their follow-up (including all appointments) in primary healthcare services over their first year of life were considered. Data collection occurred from January to November 2018.

### Population, sample, and inclusion and exclusion criteria

**Figure 1:**
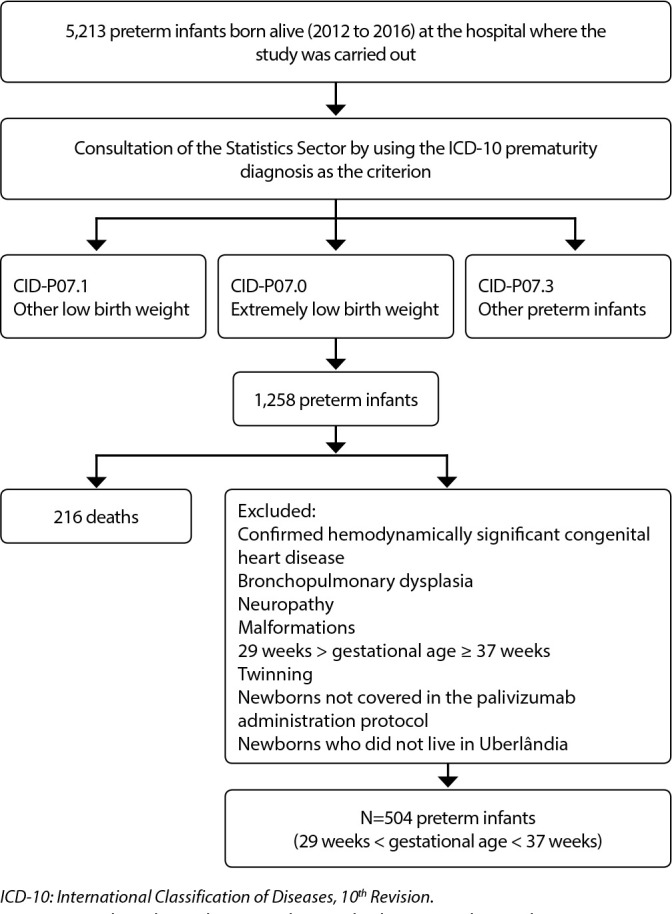
Flowchart showing the study design and population

Figure 1 shows a flowchart with the study design and population definition. There were 5,213 births of preterm infants during the study period and, by applying the concept used in the International Classification of Diseases (ICD-10), the set of preterm infants eligible for the study was narrowed down to children with GA from 29 weeks to 36 weeks and six days, which resulted in a group of 1,258 (24.1%) preterm infants. This GA was chosen because it is the interval that is not included in the PVZ use protocol^(11)^. Preterm babies with hemodynamically significant congenital heart disease, bronchopulmonary dysplasia, neuropathy, and congenital malformations, twins, and infants who died or did not live in the municipality of Uberlândia were excluded. The final sample was 504 (9.7%) preterm infants.

### Study protocol

The patients whose data were extracted were identified by consulting the Hospital Information and Statistics Sector of the hospital. The inclusion and exclusion criteria were applied. The sector provided the record number of each preterm baby born in the study period to the main researcher, who attended the hospital between January and November 2018 to access printed medical records. In order to get the primary healthcare data, the main researcher created an account on Fast Medic, a system used to manage electronic medical records in the municipal health network, so she could analyze care received by the preterm infants over their first year of life.

A data collection script was validated in 2017 by three experts, who were all nurses. One was a researcher and professor and two developed their professional activities in primary health care. The first had a PhD, and the other two had master’s degrees in public health. The average time developing research or working in the field of pediatrics in primary health care was 20 years.

The script had two parts. The first was designed to collect sociodemographic data of the babies’ mothers (age, level of education, profession, income, and parity). The second aimed to gather the preterm infants’ clinical data from birth to hospital discharge, including weight, diet type at hospital discharge, respiratory diagnosis (hospital medical records), and care received over the first year of life, including date and type of provided service, complaint, and medical diagnosis (primary healthcare medical record). The first assessment round validated the script, with 100% of agreement between the experts and no need for adjustments. The collected information was organized into electronic spreadsheets by the main researcher for posterior validation and analysis. All the medical records were consulted, and there were missing data in hospital medical records for the following variables: level of education, smoking, length of hospital stay, admission to intensive care unit, respiratory diagnosis, and diet type at hospital discharge.

### Results analysis and statistics

The World Health Organization laid the basis for the definition of ARIs. The symptoms of this class of diseases are presence of fever or not, cough and often coryza, nasal congestion, sore throat, wheezing, tachypnea, and difficulty breathing^(1)^. Occurrence of respiratory disease in the preterm infants was considered the dependent variable.

After a double data entry into Microsoft Excel software spreadsheets and data validation, the qualitative variables were analyzed over time so absolute and relative frequencies were calculated. For the quantitative variables, measures of central tendency and dispersion were obtained for each period. The number of respiratory events for each preterm infant was also calculated, and a Poisson regression model was obtained by using SAS software, version 9.4. Maternal age, level of education, smoking, pregnancy length, date of birth within the RSV season, weight at birth, and length of hospital stay were considered covariables in the model. With these data, descriptive and posterior inferential analyses were carried out in order to verify the presence of associations between independent variables related to mothers, families, and preterm infants and the dependent variable occurrence of respiratory disease. The study hypothesis was that preterm infants not included in the PVZ protocol showed a high risk of developing respiratory complications; therefore, they should be monitored and receive PVZ.

## RESULTS

In the study period, 504 preterm infants met the inclusion criteria. Their mothers were young, 348 (69.0%) were aged between 20 and 34 years old and 340 (67.6%) had over five years of formal education. Most mothers had had previous pregnancies, and 265 (52.6%) attended more than six prenatal appointments. Among the newborns, 255 (50.7%) were boys, 223 (44.2%) had GA over 35 weeks, and 385 (76.4%) had low weight at birth.

Table 1 shows that some preterm infants had no attendance records in their first year of life, whereas more than half had over six appointments in the same period. In the analyzed 1,089 appointments, the main complaint was respiratory impairment, and the highest frequency of respiratory episodes was two.

**Table 1:** Frequency of clinical variables in appointments of preterm infants over their first year of life, Uberlândia, Minas Gerais, Brazil, 2012-2016

Variable	n	%
Number of appointments		
No attendance records	97	19.2
< 6	142	28.2
≥ 6	265	52.6
Main complaint (N=1,089)		
Respiratory diseases	803	56.0
Digestive diseases	141	10.0
Nutritional and metabolic diseases	73	5.0
Ear diseases	72	5.0
Respiratory episodes (N=407)		
0	112	27.5
1	72	17.7
2	81	19.9
3	56	13.8
4	29	7.1
5	27	6.6
≥ 6	30	7.4

Physicians were the professional category in charge of nearly all the appointments (1,975; 97.2%). Appointments to deal with respiratory issues of four preterm infants stood out, two with 10 events and two with 11. Analysis of the cases of upper respiratory tract diseases indicated that 80% of the events were related to upper airway infections and acute nasopharyngitis (common cold). There were 356 preterm infants with lower respiratory tract diseases, of whom 105 (29.5%) received a bronchiolitis diagnosis. In this subgroup, 74 (20.3%) babies had one episode of the disease, 20 (5.6%) had two episodes; seven (1.9%) had the same diagnosis three times; three (0.8%) had four episodes, and one (0.3%) had bronchiolitis diagnosed five times. The disease identified in over half the episodes involving problems in the lower respiratory tract was acute viral bronchiolitis.

The data in Table 2 show that the average number of appointments over the first year of life was approximately six, a value similar to that advocated in the municipality’s child follow-up calendar. There were few sporadic appointments.

**Table 2:** Values obtained for the variables related to appointments of preterm infants over their first year of life, Uberlândia, Minas Gerais, Brazil, 2012-2016

Variable	Average	Median	Min-max	SD^†^
Appointments per preterm infant	5.75	6.0	0-20	4.49
Routine appointments	5.0	5.0	0-15	3.12
Sporadic appointments	2.13	1.0	0-12	2.35
Respiratory episodes	2.16	2.0	0-11	2.18
Bronchiolitis episodes	0.43	0	0-5	0.8

† *SD - standard deviation.*

**Figure 2:**
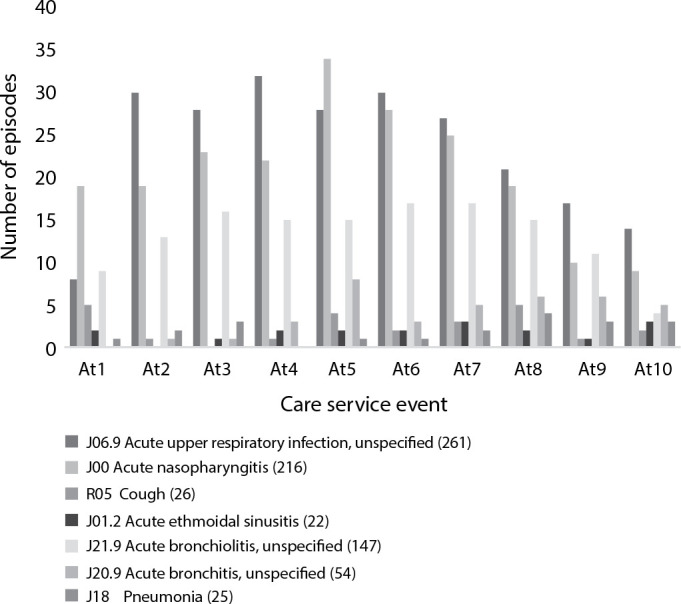
Respiratory tract medical diagnoses based on the International Classification of Diseases, 10th Revision, given during appointments of preterm infants over their first year of life, Uberlândia, Minas Gerais, Brazil, 2012-2016

Figure 2 shows the upper and lower respiratory tract disease diagnoses that were most frequent (in number of episodes) during 10 out of 20 appointments over the first year of life.

Other upper respiratory tract diseases that caused less frequent episodes and were not included in Figure 2 were: acute tonsillitis (n=12), allergic rhinitis (n=8), acute pharyngitis (n=7), laryngitis (n=4), other nose disorders (n=4), and influenza (n=1). Asthma (n=11) and laryngotracheitis (n=5) stood out among upper respiratory tract diseases. The respiratory syncytial virus is seasonal, and over the five-year period analyzed in the present study there were 1,316 (45.39%) appointments in the months during which the virus is circulating (from March to July). Hospitalization was required for 44 (3.3%), of which three were admissions to the intensive care unit. No correlation was found between maternal and infants’ variables and respiratory episodes over the first year of life (Table 3). Table 4 shows the correlation between number of sporadic appointments and number of respiratory episodes. As the number of sporadic appointments increased, the number of episodes of respiratory diseases also grew. Preterm infants who were fed exclusively on formula milk showed a higher average number of episodes of respiratory diseases at hospital discharge.

**Table 3:** Maternal and preterm infants’ variables and their correlation with episodes of respiratory disease, 2012-2016, Uberlândia, Minas Gerais, Brazil, 2018

Variable	n (%)	Estimate	Standard error	95% CI	*p* value^†^
Mothers					
Age (years)		-0.0018	0.0063	-0.0142-0.0105	0.77
≤20	95(18.8)				
20 to <35	348(69.0)				
≥35	61(12.1)				
Level of education		-0.0672	0.0828	-0.2296- 0.0951	0.417
No formal education	2(0.4)				
Middle school	161(32.0)				
High school	281(55.9)				
Higher education	59(11.7)				
Smoking		0.066	0.0997	-0.1294-0.2614	0.5082
No	418(83.4)				
Yes	3(16.6)				
Parity (pregnancies)		-0.1611	0.0855	-0.3287-0.0065	0.0596
1	198(39.3)				
≥2	306(60.7)				
Preterm infants					
Gender		0.0833	0.0715	-0.0569-0.2235	0.2443
Male	255(50.7)				
Female	248(49.3)				
Gestational age (weeks)		0.0008	0.0298	-0.0577-0.0592	0.9795
29 to 32	95(18.8)				
33 to 34	128(25.4)				
35 to 36	281(55.7)				
Weight at birth (grams)		0	0.0001	-0.0003-0.0002	0.9348
<2,500	385(76.4)				
≥2,500	119(23.6)				
Length of hospital stay§ (days)		-0.0021	0.0034	-0.0089-0.0046	0.5387
<7	234(46.7)				
7 to <14	69(13.8)				
14 to <21	51(10.2)				
≥21	147(29.3)				
Admission to intensive care unit		-0.191	0.1087	-0.404-0.022	0.0788
No	230(46.0)				
Yes	270(54.0)				
Respiratory diagnosis at hospital discharge		-0.0875	0.0833	-0.2507-0.0757	0.2931
None	200(41.3)				
At least one	284(58.7)				

† *Poisson regression model estimates. Differences to totals are explained by missing data; §Length of hospital stay from birth to discharge.*

**Table 4:** Estimates of occurrence of respiratory diseases in preterm infants taking into account birth date within RSV season, diet type at hospital discharge, and sporadic appointments over the first year of life, 2012-2016, Uberlândia, Minas Gerais, Brazil, 2018

Variable	Estimate	Standard error	95% CI	*p* value^†^
Birth date^§^	-0.1008	0.0715	-0.241-0.0393	0.1584
Diet at discharge: EB vs. FM^^*^^	-0.2355	0.1491	-0.5277-0.0567	0.1142
Diet at discharge: MB vs. FM^£^	-0.1709	0.1361	-0.4375-0.0958	0.2092
Number of sporadic appointments	0.249	0.0119	0.2257-0.2723	<0001

† *Poisson regression model estimates;* ^**§**^ Birth month within the respiratory syncytial virus season; ^^*^^Exclusive breastfeeding vs. formula milk; ^**£**^ *Mixed breastfeeding vs. formula milk.*

Analysis of the 500 hospital medical records with information about diet type at hospital discharge showed that 183 (36.6%) preterm infants were under exclusive breastfeeding (EB), 286 (57.2%) under mixed breastfeeding (MB), and 31 (6.2%) were fed on formula milk (FM). The average number of episodes of respiratory diseases for each of these groups was 2.23 (SD=2.34, median=2, minimum=0, maximum=11), 2.06 (SD=2.07, median=2, minimum=0, maximum=11), and 2.88 (SD=2.32, median=2.5, maximum=9), respectively.

The variables maternal age, hospital discharge with respiratory diagnosis, and number of sporadic appointments showed a significant association with number of episodes of bronchiolitis (Table 5).

**Table 5:** Poisson regression model estimates with the variable number of episodes of bronchiolitis as the answer, Uberlândia, Minas Gerais, Brazil, 2018

Parameter	Estimate	Standard error	95% CI		*p* value^†^
Intercept	0.3924	2.3639	-4.2408	5.0255	0.8682
Maternal age	-0.0345	0.0167	-0.0672	-0.0017	0.039
Birth date§	0.1025	0.1748	-0.2402	0.4452	0.5577
Level of education	-0.0423	0.1985	-0.4314	0.3469	0.8314
Smoking	0.3447	0.2316	-0.1093	0.7986	0.1367
Gestational age	-0.0349	0.0705	-0.1731	0.1033	0.6205
Length of hospital stay	-0.0016	0.0082	-0.0177	0.0145	0.8492
Weight at birth	-0.0001	0.0003	-0.0007	0.0005	0.6917
Childbirth and pregnancy	-0.3161	0.2093	-0.7263	0.0942	0.131
Respiratory diagnosis at discharge	-0.4398	0.2002	-0.8323	-0.0474	0.028
Diet at discharge: EB vs. FM^*^	0.4554	0.4139	-0.3559	1.2668	0.2712
Diet at discharge: MB vs. FM^£^	0.4876	0.3909	-0.2785	1.2537	0.2122
Admission to intensive care unit^‡^	-0.3064	0.263	-0.8218	0.209	0.244
Number of sporadic appointments	0.2647	0.0295	0.207	0.3225	<.0001
Gender	0.3352	0.1754	-0.0085	0.6789	0.056

† *Poisson regression model estimates;* ^**§**^ Month of birth within the respiratory syncytial virus season; ^^*^^Exclusive breastfeeding vs. formula milk; ^**£**^ *Mixed breastfeeding vs. formula milk.*

## DISCUSSION

Preterm infants with GA between 29 weeks and 37 incomplete weeks who were not covered by the PVZ protocol were at risk of developing respiratory complications. Studies have shown that newborns with GA higher than 29 weeks had lower respiratory tract diseases and could be hospitalized because of them and that one of the factors that increased this risk was using formula milk^(9,15)^. The finding of the present study that preterm infants fed exclusively on formula milk at hospital discharge had a higher frequency of respiratory episodes confirmed this result.

The analyzed preterm infants had five appointments over their first year of life and slightly more than two diagnoses of respiratory impairment on average, a number lower than four to eight episodes of acute respiratory infection, the range mentioned by the Pan American Health Association. This number was reported to reach 10 occurrences per year in children that attended daycare centers^(16)^. Every year, the rate gets closer to the numbers cited in the Pneumonia Brazilian Guidelines, that is, from four to six acute respiratory infections in most children^(16)^. However, a cohort study^(17)^ found an incidence of 1.8 episodes of acute respiratory infections in breastfed full-term newborns, and RSV was the most identified virus in lower respiratory tract diseases.

Use of PVZ was approved in late 1990s, and the Committee on Infectious Diseases at the American Academy of Pediatrics recommended immunoprophylaxis of breastfed infants that showed increased risk of developing an infection with RSV. The recommendations were revised in 2003, 2009, and 2014^(18)^. The last revision established the offering of PVZ to preterm infants with GA lower than 29 weeks and before 12 months of age in case the date of birth is at the beginning of the virus season. Newborns with GA between 29 and 34 weeks with no extra risk factor involving serious illness caused by RSV and those with GA higher than 32 weeks were excluded from the group of infants that were eligible for receiving this monoclonal antibody^(18-19)^.

The change in the guidelines about prophylactic use of PVZ led to a risk of developing an infection with RSV 55% higher for newborns with GA between 29 and 32 weeks in comparison with the probability calculated for full-term babies^(18)^. The severity of the condition increased in preterm infants, with worse complications in those younger than three months^(18)^. Another study identified a marked increase in the occurrence of hospitalizations in children younger than 90 days and the death of one baby per season^(20)^.

Results reported in a systematic review^(20)^ that examined nearly 400 publications by considering that guidelines for PVZ use should be in accordance with the most recent evidence recommended administration of PVZ to preterm infants with GA lower than 29 weeks and lower than or equal to 31 weeks and nine or six months of age during the virus season; high-risk children with GA from 32 to 35 weeks and less than two years old showing bronchopulmonary dysplasia or significant congenital heart disease; and high-risk populations, such as children with Down syndrome, lung or neuromuscular disorders, cystic fibrosis, and immunocompromised children.

The last update in the American Academy of Pediatrics recommendations, in 2014, limited use of PVZ to breastfed babies with GA lower than 29 weeks and children younger than one year old. Therefore, preterm infants with GA from 29 to 34 weeks are no longer eligible for this prophylaxis^(19)^. A study analyzed the impact of the updated orientation in the only American center that had the cause of hospitalizations confirmed by laboratory tests and concluded that there was an increase in the number of hospitalizations caused by infections with RSV and higher morbidity of the breastfed babies with GA from 29 to 34 weeks over the year that followed the implementation of the new guidelines regarding prophylaxis with PVZ^(19)^.

In contrast with the findings of the present study, a significant association between hospitalization caused by lower respiratory tract disease, male gender, maternal age lower than 25 years, and dependence on oxygen after birth was found^(21)^. A prospective cohort study^(22)^ showed similar average maternal age; however, variables such as maternal age and level of education and existence of siblings with acute respiratory infection showed no association with hospitalization, similar to what was found in the present study. Nevertheless, the results differed regarding another variable: there was an association between low weight at birth and occurrence of pneumonia. Children with low weight at birth (<2,500 g) had chances of having pneumonia episodes approximately six times higher in comparison with the probability calculated for babies with adequate weight at birth^(23)^. It is important to consider these aspects, since a systematic review^(23)^ that verified risk factors for hospitalization of healthy preterm children as a consequence of infection with RSV highlighted lower age within the virus season and existence of a school-age sibling as relevant factors.

Authors showed that, in Italy^(5)^, there were 1.6 times more episodes of bronchiolitis in boys. Another study^(7)^, which found an association between age at first severe infection with RSV and age at the first severe episode of asthma also indicated a higher prevalence in male children; however, variables such as smoking during pregnancy, multiparity, and vaginal birth showed differences compared with the findings of the present study. The authors also verified higher occurrence of consecutive asthma in children with severe respiratory disease after six months of life in comparison with the rate obtained for children with episodes before this age^(7)^.

The present study found data about hospitalizations and children who required critical care that corroborated previous findings^(3,24)^. A systematic review^(6)^ indicated that a considerable proportion of morbidity in children over the first year of life was associated with RSV, especially in preterm infants, and emphasized the relevance of the illness caused by this virus as a cause of hospitalization and its contribution to these children’s mortality, with GA being a critical determinant of the severity of the disease^(6)^.

Still regarding the results of the study developed in Italy^(5)^, almost 5% of the newborns were hospitalized with a diagnosis of bronchiolitis over the first year of life. Babies with GA from 33 to 34 weeks showed a hospitalization rate twice as high as that observed for newborns with GA superior to 35 weeks. A study in Eastern Europe^(21)^ showed that, within the virus season, the incidence of hospitalizations of late preterm infants caused by lower respiratory tract diseases was 6.5%, whereas the number for lower respiratory tract infections caused by RSV was 1.7%.

Analysis of the preterm infants’ diet indicated that those who were given exclusively FM at hospital discharge had more respiratory episodes over their first year of life. A study^(25)^ with 113 preterm babies assessed the incidence of EB and the factors associated with its discontinuation after hospital discharge in a Brazilian capital. The results for the variables maternal age, birth type, average GA, baby gender, critical care duration, and use of ventilatory support were similar to those found in the present study. The incidence of EB at discharge, 81.4%, differed from that obtained in the present study, but was substantially lower on the second week at home. There was an association between higher weight at birth, double pregnancy, shorter period on mechanical ventilation (invasive and noninvasive), and a higher risk of discontinuing EB^(25)^.

An association between variables such as pre, peri, and postnatal conditions and hospitalizations caused by bronchiolitis over the first year of life was also identified in another study^(5)^. Regarding neo and perinatal risk factors, the data indicated an association between first pregnancy, previous use of surfactant by preterm infants, absence of breastfeeding, and hospital admission caused by bronchiolitis. Environmental factors, existence of siblings younger than 10 years old, agglomeration, and exposure to the virus during its season were mentioned as postnatal conditions^(5)^.

The findings of the present study emphasized the importance of focusing on surveillance actions, especially in primary health care, to develop improvements in care of children under five years old and seek care comprehensiveness, with the understanding of this population’s specificities as a starting point^(26-27)^. Although most appointments were programmed, approximately one third of the appointments were sporadic and statistically significant for respiratory disease as an event.

Programmed appointments over the first year of life are convenient opportunities to offer health care, with the possibility of identification of vulnerabilities in a unique period of a child’s life, especially when it is a preterm infant. In these occasions, care actions such as evaluation of growth and development, support to and encouragement of EB and eating^(13-14,26-28)^, guidance on immunization schedule, and help with questions and uncertainties mothers may have regarding basic care of their children are imperative^(27-28)^.

In Brazil, primary health care, instituted by public policies, is the healthcare segment responsible for following up every child since birth. However, preterm infants who undergo extended hospitalization right after birth require constant and effective follow-up^(13-14,27)^ once they are discharged. If possible damage is identified, timely care must be immediately offered. In this context, an extremely important health action is home visit, carried out by nursing teams on the first week after discharge. This is an essential tool, since it allows the development of a bond and the evaluation of the child’s adaptation to the environment, which is especially necessary when it is a high-risk one^(26-28)^.

It is important to emphasize the responsibility of health professionals, including nurses, for these children: their care actions must focus on early detection and timely treatment of diseases that prevail in this stage of life^(27-28)^. They must also identify high-risk children and carry out an active search for patients who do not attend routine appointments^(26-28)^. Last, it must be stressed that preterm infants who were fed exclusively on FM at hospital discharge had a higher average number of respiratory disease episodes. Additionally, EB proved to be a protective factor, as previously shown in other studies^(13-14)^.

### Study limitations

The main limitation of the present study was collection of data from medical records that had incomplete information, which hindered generalization of the results.

### Contributions to the nursing, health, or public policy area

Although it was not possible to generalize the findings, the present study delimited specificities of preterm infants who did not receive PVZ and had respiratory disease episodes over the first year of life. Teams that deliver care to these children must constantly reinforce actions oriented toward promoting breastfeeding. Given the proximity nurses have with families, they are the professionals who play an indispensable role in providing guidance during child care appointments, which fell short of the recommendations in the analyzed municipality.

Absence of breastfeeding at hospital discharge and sporadic appointments and their relationship with the outcome development of respiratory disease proved to have great implications in nursing clinical practice. Both during hospital stay and primary health care delivery, nurses must put into practice actions that strengthen the initiation of breastfeeding in preterm infants and maintain its continuity.

Given the high cost of PVZ, the results of the present study indicated that identifying high-risk preterm infants that are not included in the PVZ use protocol and encouraging and promoting EB are feasible strategies that can minimize the occurrence of respiratory diseases and their future complications.

## CONCLUSIONS

Exclusive use of FM at hospital discharge and number of sporadic appointments resulted in more respiratory episodes in preterm infants who did not receive PVZ, and breastfeeding proved to have a protective effect. These findings indicated the need to individualize care of this population. Preparation for discharge and coordination of care for subsequent appointments in primary care are elements that have to be considered by health teams. Analysis of bronchiolitis cases in preterm infants emphasized how indispensable timely care delivery is to sort out complications that can lead to hospital admission. Identifying preterm infants whose diet is based on FM at hospital discharge is relevant so measures to prevent respiratory diseases can be taken.

Strategies in primary health care that make a close contact between health teams and preterm infants and their families feasible, such as home visits, appointments over the first year of life, and support to and encouragement of breastfeeding are powerful tools to minimize hospitalization episodes, need for intensive care, and complications resulting from respiratory diseases in preterm infants who are not included in the PVZ administration protocol.
